# Alterations in the Mineral Bone Metabolism of Living Kidney Donors After Uni-Nephrectomy: Prospective Observational Study

**DOI:** 10.3389/fmed.2021.741944

**Published:** 2021-10-15

**Authors:** Hanbi Lee, Sang Hun Eum, Eun Jeong Ko, Hyuck Jin Cho, Chul Woo Yang, Byung Ha Chung

**Affiliations:** ^1^Division of Nephrology, Department of Internal Medicine, Seoul St. Mary's Hospital, The Catholic University of Korea, Seoul, South Korea; ^2^Transplant Research Center, Convergent Research Consortium for Immunologic Disease, College of Medicine, The Catholic University of Korea, Seoul, South Korea; ^3^Department of Urology, Seoul St. Mary's Hospital, The Catholic University of Korea, Seoul, South Korea

**Keywords:** chronic kidney disease-mineral and bone disorder, living donors, nephrectomy, phosphate, patient safety

## Abstract

We investigated the dynamic change of mineral bone metabolism and explored factors associated with the alteration of mineral bone metabolism in the living kidney donors (LKDs) after uni-nephrectomy. One-hundred forty-four prospective LKDs who underwent kidney donation between May 2016 and September 2018 were enrolled. Laboratory evaluation regarding mineral bone metabolism including intact parathyroid hormone (iPTH), renal fractional excretion of phosphate (FEPi), and technetium-99m diethylenetriaminepentaacetate (^99m^Tc-DTPA) scan was performed predonation and 6 months after donation. We divided donors into two groups, the low ΔFEPi and high ΔFEPi groups, according to the change of FEPi after donation, and investigated significant risk factors associated with high ΔFEPi. At 6 months after uni-nephrectomy, estimated glomerular filtration rate (eGFR) significantly declined by 30.95 ml/min/1.73 m^2^ (*p* < 0.001), but the measured GFR (mGFR) of the remaining kidney by ^99m^Tc-DTPA scan showed significant increase. Serum phosphorus decreased (*p* < 0.001), whereas FEPi (13.34–20.23%, *p* < 0.001) and serum iPTH (38.70–52.20 pg/ml, *p* < 0.001) showed significant increase. In the high ΔFEPi group, the proportion of preexisting hypertension (HTN) was higher, the baseline FEPi was lower, and the percent decline in eGFR was greater. Moreover, all of these factors were independently associated with high ΔFEPi upon multivariable logistic regression analysis. LKDs showed a significant change in mineral bone metabolism after uni-nephrectomy, especially when the donors had preexisting HTN, lower baseline FEPi, and showed greater loss of kidney function. Hence, strict monitoring of the mineral bone metabolism parameters and bone health may be required for these donors.

## Introduction

It is well known that most patients with chronic kidney disease (CKD) show a progressive deterioration in mineral homeostasis. These changes have been associated with a complex state termed CKD-mineral and bone disorder (CKD-MBD), which can exhibit fragility fractures, vascular calcification, and cardiovascular disease or mortality (1–4). As the renal function declines, the ability of the kidneys to appropriately excrete a phosphate load is reduced leading to hyperphosphatemia, elevated parathyroid hormone (PTH), and decreased calcitriol with the associated elevations in the levels of fibroblast growth factor-23 (FGF-23) (5, 6). Although CKD-MBD begins early in CKD, serum phosphate level is normal due to compensatory increase in FGF-23 and PTH at the early stage. In this situation, renal fractional excretion of phosphate (FEPi) was reported to be a useful marker of phosphate homeostasis, an alternative to FGF-23 (7).

Meanwhile, the mechanisms of mineral bone metabolism adaptation in response to a loss of glomerular filtration rate (GFR) in early CKD are still controversial (8). Kidney donation allows us to determine the direct effect of a mild reduction in GFR on mineral bone metabolism without the other confounding factors (9). One of the largest prospective donor studies, the Assessing Long Term Outcomes in Living Kidney Donors (ALTOLD) study, compared the kidney donors to a paired control group, showing that mineral bone metabolism adaptation occurred after donation (10). However, the prospective change of the parameters associated with CKD-MBD and the significant factors associated with the progression of CKD-MBD have not yet been fully investigated.

In this regard, we conducted a prospective observational study of the living kidney donors (LKDs) to investigate whether uni-nephrectomy for kidney donation causes a change of mineral bone metabolism and explore which factors are associated with mineral bone metabolism adaptation by using FEPi as a surrogate marker for phosphate regulation in CKD.

## Patients And Methods

### Study Population

A total of 280 carefully screened healthy LKDs who underwent uni-nephrectomy for kidney donation in Seoul St Mary's Hospital (Republic of Korea) between May 2016 and September 2018 were enrolled prospectively. Participants were enrolled after acceptance for donation, but before donation had taken place. Participants who did not perform 24-h urine collection or technetium-99m diethylenetriaminepentaacetate (^99m^Tc-DTPA) scintillation-camera renography in two subsequent time points, predonation and 6 months after donation were excluded. Finally, out of 280 LKDs, 144 participants completed the studies after kidney donation and they were included in the postdonation analysis. This study was approved by the Institutional Review Board of Seoul St Mary's Hospital (KC14ONMI0460).

### Clinical and Laboratory Parameters

We collected demographic and clinical data such as age at kidney donation, sex, preexisting hypertension (HTN), body mass index, and donated kidney laterality. Laboratory evaluation, 24-h urine collection, and ^99m^Tc-DTPA were performed in two subsequent time points, predonation and 6 months after donation. GFR was estimated by using the CKD-Epidemiology Collaboration (CKD-EPI) equation based on serum creatinine (11). Serum calcium was corrected for albumin according to the following formula: total calcium (mg/dl) + 0.8 × (4.0 − serum albumin (g/dl)) (if serum albumin < 4.0 g/dl). Serum intact PTH (iPTH) and 25-hydroxyvitamin D were measured by chemiluminescence immunoassay. FEPi was calculated as $\frac{\left[\text{urine phosphorus }\left(\text{mg}/\text{dl}\right)\;\times \text{ serum creatinine }\left(\text{mg}/\text{dl}\right)\;\times \;100\right]}{\left[\text{serum phosphorus }\left(\text{mg}/\text{dl}\right)\;\times \text{ urine creatinine }\left(\text{mg}/\text{dl}\right)\right]}$ (12). Calcium-phosphorus product concentration was calculated as *serum calcium* (*mg*/*dl*) × *serum phosphorus* (*mg*/*dl*). Serum intact FGF-23 (Millipore Corporation, Billerica, Massachusetts, United States of America) was measured by using the ELISA according to the protocol of the manufacturer. Due to the wide variation of FGF-23 values, we performed analysis after the semi-log transformation of the data. According to the method described by Russel et al. (13), GFR was measured (mGFR) by ^99m^Tc-DTPA scintillation-camera renography, which is regarded as the gold standard of measuring split kidney function (14) with a single injection technique by using a four-point sampling approach at 10, 30, 180, and 240 min after injection. Renal angio 3D CT images were used for reconstruction and imported to a volume measuring software, AquariusNET Thin Client (TeraRecon, Inc., San Mateo, CA) to calculate the renal volumes. Renal volume and GFR were adjusted for body surface area and calculated by using the Du Bois and Du Bois method (15).

We calculated ΔFEPi by the difference of FEPi between baseline and 6 months after the kidney donation. Since the difference of FEPi was normally distributed, we used the mean value of ΔFEPi (7.41 ± 6.16%) as the cutoff for dividing the participants into the low ΔFEPi group (*n* = 76) and high ΔFEPi group (*n* = 68). We compared the clinical characteristics between the low ΔFEPi and high ΔFEPi groups and evaluated the significant risk factors associated with high ΔFEPi.

### Statistical Analysis

All the continuous variables were expressed as the mean ± SD or median [interquartile range (IQR)]. The changes of parameters between two time points, predonation and 6 months after kidney donation, were evaluated by the paired comparison. If the variables followed the normal distribution, paired *t*-test was performed; otherwise, the Wilcoxon signed-rank test was performed. All the categorical variables were compared by using the chi-squared test or Fisher's exact test and expressed as proportions. In the comparisons of the two groups, the Student's *t*-test or Mann–Whitney U test was performed. The predictors of the greater mineral bone metabolism alterations were explored with multivariable logistic regression analysis. Baseline clinical and laboratory parameters showing the significant differences (*p* < 0.05) in univariable analysis or known to affect mineral bone metabolism alterations that were fitted into the multivariable model. We selected age, sex, preexisting HTN, predonation FEPi, and percent decline in estimated GFR (eGFR) as the predictors. Two-sided *p* < 0.05 were considered as statistically significant. All the statistical analyses were performed by using the SPSS® software version 24 (IBM Corporation, Armonk, New York, USA) and GraphPad Prism version 8.4.3 (686) (GraphPad Software, San Diego, CA, USA).

## Results

### Baseline Characteristics of the Living Kidney Donors

The baseline characteristics of the cohort are presented in Table 1. The mean age was 44.80 ± 11.96 years, 50 (34.7%) were males, and 11 (7.6%) had preexisting HTN. The mean predonation eGFR was 99.32 ± 12.88 ml/min/1.73 m^2^. None of the LKDs in our cohort had predonation eGFR of <60 ml/min/1.73 m^2^ and 36 (25.0%) of them had predonation eGFR of 60–89 ml/min/1.73 m^2^. Serum creatinine, corrected calcium, phosphorus, 25-hydroxyvitamin D, intact PTH, and 24-h urine protein were within the reference range. At baseline, the mean FEPi was 14.13 ± 4.70%.

**Table 1:** Baseline characteristics of the living kidney donors.

	**Results**	**References**
**Demographics**		
Age (years)	44.80 ± 11.96	
Male (*n*, %)	50, 34.7%	
HTN (*n*, %)	11, 7.6%	
Body mass index (kg/m^2^)	23.56 ± 3.21	
Lt. kidney donation (*n*, %)	116, 80.6%	
Adjusted dk-mGFR (ml/min/1.73m^2^)	50.02 ± 8.66	
Adjusted rk-mGFR (ml/min/1.73m^2^)	54.56 ± 10.22	
Adjusted dk-volume (cm^3^/1.73m^2^)	173.87 [157.68–193.09]	
Adjusted rk-volume (cm^3^/1.73m^2^)	171.56 [155.25–193.00]	
**Biochemical parameters**		
Serum creatinine (mg/dl)	0.79 ± 0.14	0.6~1.2
eGFR (CKD-EPI) (ml/min/1.73m^2^)	99.32 ± 12.88	
Alkaline phosphatase (U/L)	50.26 ± 13.29	40~129
Serum albumin (g/dl)	4.39 ± 0.25	3.5~5.2
Serum corrected calcium (mg/dl)	9.19 ± 0.37	8.0~10.0
Serum phosphorus (mg/dl)	3.59 ± 0.48	2.6~4.5
25-hydroxyvitamin D (ng/ml)	24.43 ± 9.26	
Intact PTH (pg/ml)	42.18 ± 18.67	14~72
24-h urine protein (mg/day)	36.00 [20.00–62.50]	
24-h FEPi (%)	14.13 ± 4.70	

*Continuous variables are presented as mean ± SD or median (interquartile range). Categorical variables are presented as proportions (%)*.

*HTN, hypertension; dk-mGFR, measured glomerular filtration rate of the donated kidney; rk-mGFR, mGFR of the remaining kidney; dk-volume, the volume of the donated kidney; rk-volume, the volume of the remaining kidney; eGFR, estimated GFR; PTH, parathyroid hormone; FEPi, renal fractional excretion of phosphate*.

The left kidney was selected and procured for LKD transplantation in 116 (80.6%) donors. There was a statistically significant difference between mGFR of the donated kidney (dk-mGFR) (50.02 ± 8.66 ml/min/1.73 m^2^) and mGFR of the remaining kidney (rk-mGFR) (54.56 ± 10.22 ml/min/1.73 m^2^) (*p* < 0.001). On the other hand, no significant difference of adjusted volume between the donated kidney (dk-volume) (173.87, IQR: 157.38–193.09 cm^3^/1.73 m^2^) and the remaining kidney (rk-volume) (171.56, IQR: 155.25–193.00 cm^3^/1.73 m^2^) was observed (*p* = 0.651).

### Change of Renal Function and the Biochemical Parameters of Mineral Bone Disease After Uni-Nephrectomy

**Figure 1:**
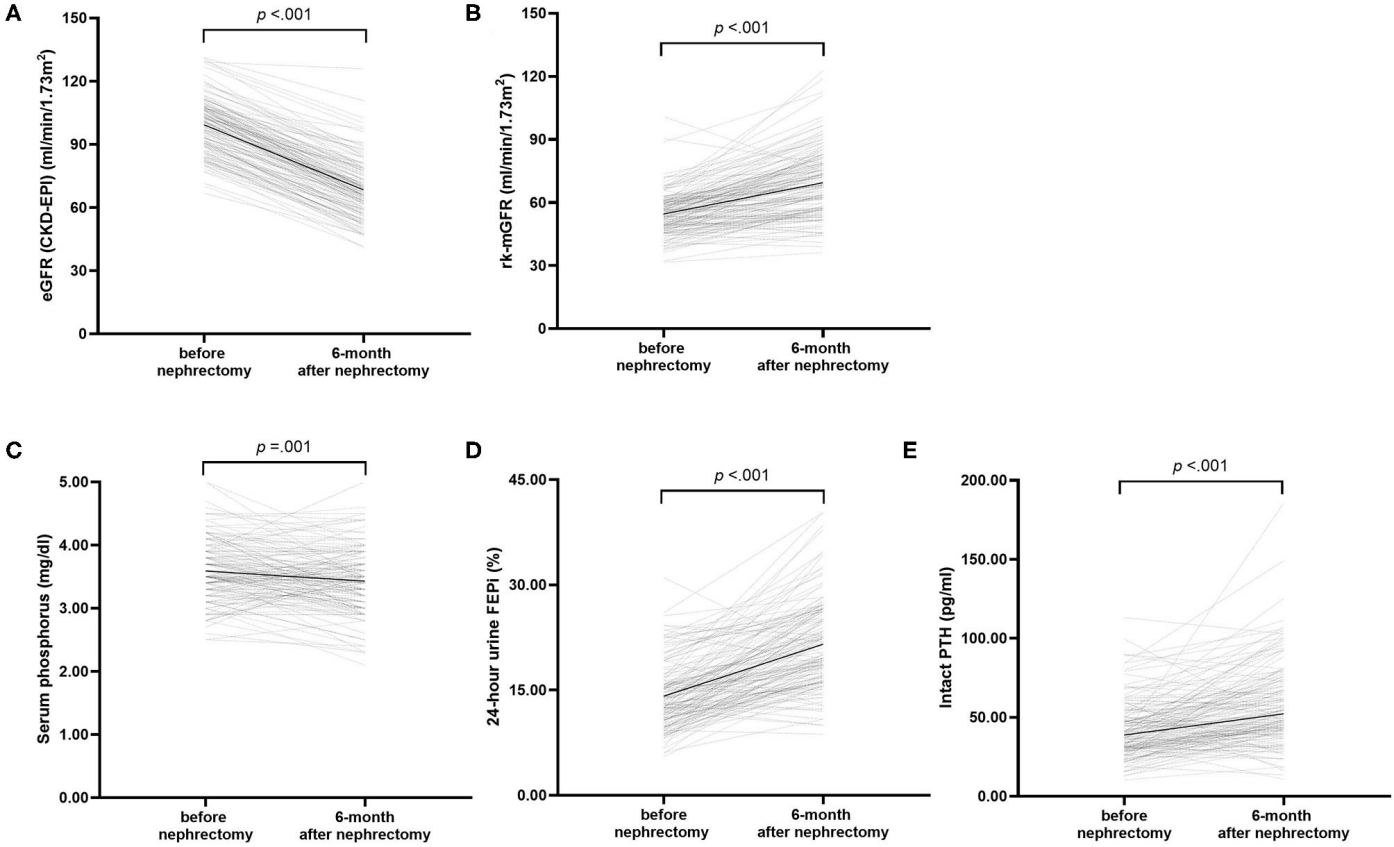
Changes in renal function and the biochemical parameters after donation. **(A)** estimated glomerular filtration rate (GFR), **(B)** measured GFR of the remaining kidney, **(C)** serum phosphorus concentration, **(D)** 24 h urine renal fractional excretion of phosphate, and **(E)** serum intact parathyroid hormone concentration. Black lines illustrated mean or median. *p*-values compare before after nephrectomy by paired *t*-test.

The follow-up interval after uni-nephrectomy was within a range of 5.52 months and 7.96 months. At 6 months after uni-nephrectomy, we observed a statistically significant decrease of 30.95 ml/min/1.73 m^2^ (95% CI: 29.57–32.33) in eGFR from 99.32 ± 12.9 to 68.38 ± 13.91 ml/min/1.73 m^2^ (*p* < 0.001) (Figure 1A). We also observed a statistically significant increase of 14.87 ml/min/1.73 m^2^ (95% CI: 12.70–17.04) following donation of the adjusted rk-mGFR from 54.56 ± 10.22 to 69.43 ± 16.16 ml/min/1.73 m^2^ (*p* < 0.001) (Figure 1B). The Wilcoxon signed-rank test indicated a significant difference of 24-h urine protein between before donation (36.00, IQR: 20.00–62.50 mg/day) and after donation (52.50, IQR: 30.00–94.00 mg/day) (*p* < 0.001).

Regarding the biochemical parameters of MBD, serum phosphorus declined by 0.16 mg/dl (95% CI: 0.07–0.24) from 3.59 ± 0.48 to 3.43 ± 0.52 mg/dl (Figure 1C). The decline was significant according to a dependent *t*-test (*p* < 0.001). The increase of corrected serum calcium concentration was significant by 0.10 mg/dl (95% CI: 0.04–0.16) (*p* = 0.003), whereas the calcium-phosphorus product was significantly declined by 1.08 mg^2^/dl^2^ (95% CI: 0.22–1.95) (*p* = 0.014). FEPi was significantly increased by 7.4% (95% CI: 6.40–8.43) from 14.13 ± 4.70 to 21.54 ± 6.26% (*p* < 0.001) (Figure 1D). On average, serum iPTH was much higher after donation (52.20, IQR: 42.30–71.05 pg/ml) compared than before donation (38.70, IQR: 29.83–50.58 pg/ml) according to the Wilcoxon signed-rank test (*p* < 0.001) (Figure 1E), whereas 25-hydroxyvitamin D showed no significant difference before donation (24.43 ± 9.26 ng/ml) and after donation (23.87 ± 9.37 ng/ml) (*p* = 0.488) (Table 2).

**Table 2:** Biochemical parameters before and 6 months after uni-nephrectomy in the living kidney donors.

	**Living kidney donor (*****n*** **=** **144)**		***p*-value**
	**Before nephrectomy**	**6-month after nephrectomy**	
Serum creatinine (mg/dl)	0.79 ± 0.14	1.12 ± 0.21	<0.001
eGFR (CKD-EPI) (ml/min/1.73m^2^)	99.32 ± 12.9	68.38 ± 13.91	<0.001
Adjusted rk-mGFR (ml/min1.73m^2^)	54.56 ± 10.22	69.43 ± 16.16	<0.001
24-h urine protein (mg/day)	36.00 [20.00–62.50]	52.50 [30.00–94.00]	<0.001
Serum alkaline phosphatase (U/L)	50.26 ± 13.29	49.16 ± 12.01	0.089
Serum corrected calcium (mg/dl)	9.19 ± 0.37	9.28 ± 0.35	0.003
Serum phosphorus (mg/dl)	3.59 ± 0.48	3.43 ± 0.52	<0.001
Calcium-phosphorus product (mg^2^/dl^2^)	32.95 ± 4.86	31.86 ± 5.16	0.014
24-h urine FEPi (%)	14.13 ± 4.70	21.54 ± 6.26	<0.001
Intact PTH (pg/ml)	38.70 [29.83–50.58]	52.20 [42.30–71.05]	<0.001
25-hydroxyvitamin D (ng/ml)	24.43 ± 9.26	23.87 ± 9.37	0.488

*Continuous variables are presented as mean ± SD or median (interquartile range)*.

*eGFR, estimated glomerular filtration rate; rk-mGFR, measured GFR of the remaining kidney; FEPi, renal fractional excretion of phosphate; PTH, parathyroid hormone*.

### Comparison Between the Low Delta FEPi and High Delta FEPi Groups

We divided LKDs into two groups according to the change of FEPi after uni-nephrectomy, a low ΔFEPi group (*n* = 76) and a high ΔFEPi group (*n* = 68). Between the two groups, age, the proportion of the male participants, and body mass index (BMI) were not different. Adjusted dk-volume, adjusted dk-mGFR, and percent of dk-mGFR were similar between the two groups. A significantly high proportion of the LKDs had HTN before donation in the high ΔFEPi group than the low ΔFEPi group (2.6 vs. 13.2%, *p* = 0.017). Predonation FEPi was significantly lower in the high ΔFEPi group (14.52, IQR: 12.00–18.87 vs. 12.08, IQR: 9.68–15.19%, *p* < 0.001). Percent decline in eGFR after uni-nephrectomy was significantly higher in the high ΔFEPi group (*p* = 0.043) (Table 3).

**Table 3:** Comparison of the clinical characteristics and biochemical parameters according to the difference of FEPi after uni-nephrectomy.

	**Living kidney donor (*****n*** **=** **144)**		***p*-value**
	**Low-delta FEPi (*n* = 76)**	**High-delta FEPi (*n* = 68)**	
Age	44.39 ± 12.30	45.25 ± 11.63	0.670
Male (*n*, %)	29, 38.2%	21, 30.9%	0.360
HTN (*n*, %)	2, 2.6%	9, 13.2%	0.017
Body mass index (kg/m^2^)	23.38 ± 3.24	23.76 ± 3.19	0.484
Adjusted dk-volume (cm^3^/1.73m^2^)	174.14 [159.67–192.62]	168.65 [153.03–193.09]	0.255
Adjusted mGFR (ml/min/1.73m^2^)	103.34 ± 17.00	99.20 ± 15.93	0.135
Adjusted dk-mGFR (ml/min/1.73m^2^)	51.09 ± 8.75	48.83 ± 8.47	0.118
% of dk-mGFR	47.93 ± 3.70	47.86 ± 4.00	0.914
Serum creatinine (mg/dL)	0.79 ± 0.15	0.78 ± 0.14	0.674
eGFR (CKD-EPI) (ml/min/1.73m^2^)	99.66 ± 13.55	98.95 ± 12.18	0.742
Serum alkaline phosphatase (U/L)	48.00 [38.00–56.75]	49.00 [42.25–59.75]	0.236
Serum corrected calcium (mg/dl)	9.20 [8.90–9.40]	9.20 [8.90–9.40]	0.847
Serum phosphorus (mg/dl)	3.59 ± 0.46	3.58 ± 0.51	0.970
Intact PTH (pg/ml)	39.20 [29.98–52.35]	38.20 [29.20–49.68]	0.688
25-hydroxyvitamin D (ng/ml)	23.76 [19.08–29.86]	22.67 [18.86–29.68]	0.738
24-h urine protein (mg/day)	35.50 [19.00–56.50]	40.50 [20.25–67.50]	0.526
24-h FEPi (%)	14.52 [12.00–18.87]	12.08 [9.68–15.19]	<0.001
Percent decline in eGFR (%)	30.65 [23.96–37.03]	34.24 [28.41–38.16]	0.043
Adjusted rk-mGFR gain (ml/min/1.73m^2^)	14.50 ± 11.77	15.28 ± 14.62	0.722
Change in FEPi (%)	3.90 [1.47–5.51]	11.60 [9.51–14.51]	<0.001

*Continuous variables are presented as mean ± SD or median (interquartile range). Categorical variables are presented as proportions (%)*.

*FEPi, renal fractional excretion of phosphate; HTN, hypertension; dk-volume, the volume of the donated kidney; dk-mGFR, measured glomerular filtration rate of the donated kidney; eGFR, estimated GFR; PTH, parathyroid hormone; rk-mGFR, measured GFR of the remaining kidney*.

### Factors Associated With Mineral Bone Metabolism Disturbance After Uni-Nephrectomy

To investigate the determinants of the mineral bone metabolism disturbance after uni-nephrectomy, a multivariable logistic regression analysis was conducted. The predictor variables were tested to verify that there was no multicollinearity. Three predictor variables—percent decline in eGFR, predonation FEPi, and preexisting HTN—were found to contribute to the model (Table 4).

**Table 4:** Predictors of the alteration of mineral bone metabolism after uni-nephrectomy.

**Predictors**	**β coefficient**	**Standard error**	**Odds ratio (95% CI)**	***p*-value**
Age	−0.007	0.017	0.993 (0.961–1.027)	0.686
**Sex**				
Male	Reference			
Female	0.352	0.408	1.421 (0.638–3.165)	0.389
**Pre-existing HTN**				
No	Reference			
Yes	2.116	0.924	8.296 (1.357–50.705)	0.022
Pre-donation FEPi (%)	−0.160	0.047	0.852 (0.777–0.934)	<0.001
Percent decline in eGFR (%)	0.056	0.024	1.057 (1.008–1.109)	0.023

*Multivariable logistic regression analysis was used (n = 144)*.

An increase in percent decline in eGFR (%) was associated with an increase in the odds ratio (OR) of the progression of the alteration of the mineral bone metabolism (OR 1.051, 95% CI: 1.002–1.103, *p* = 0.042). The presence of HTN before the donation was associated with the progression of the disturbance of the mineral bone metabolism (OR 7.302, 95% CI: 1.240–43.012, *p* = 0.028). Predonation FEPi was also inversely related with the progression of the alteration of the mineral bone metabolism (OR 0.847, 95% CI: 0.773–0.928, *p* < 0.001).

## Discussion

In this study, we described the postdonation change of the renal function and mineral bone metabolism parameters and investigated the determinants of mineral bone metabolism alteration after uni-nephrectomy. We found that the percent decline in eGFR following uni-nephrectomy was less than the mean percent of dk-mGFR due to functional gain of the remaining kidney. After donation, the changes in the biochemical parameters with respect to MBD were similar to secondary hyperparathyroidism with an increase of iPTH and phosphaturia. Preexisting HTN, predonation FEPi, and percent decline in eGFR were the significant predictors of a greater increase in FEPi, a surrogate marker of mineral bone metabolism disturbance after donation.

According to the Kidney Disease: Improving Global Outcomes (KDIGO) guideline (16), none of the donors had a predonation eGFR of <60 ml/min/1.73 m^2^ in our cohort. Whether preexisting HTN would be a risk factor of accelerating the rise in blood pressure (BP) and might cause target organ damage to the remaining kidney is controversial (17). Thus, a few donors with preexisting HTN, who controlled BP using one or two antihypertensive agents without target organ damage, were observed in our cohort. Left kidney nephrectomy is favored over right kidney nephrectomy due to the relative technical ease of operation due to the longer renal vein (18). Most donor kidneys (80.3%) were procured from the left side as expected. The reason for significantly higher mGFR in the remaining kidney than in the donated kidney is the preferential selection of kidneys with lesser function for donation.

Due to functional gain of the remaining kidney in the early postdonation period, the percent decline in eGFR following uni-nephrectomy was 31.45 ± 8.23% rather than the mean percent of dk-mGFR (47.90 ± 3.83%), which is associated with the amount of loss of nephron. The results are consistent with the previous studies showing that rapid compensatory hyperfiltration of the remaining kidney leads to a net reduction in GFR of approximately 30% after donation, despite a renal mass loss of approximately one-half (16, 19–21). The kidney has a substantial functional reserve capacity to preserve renal function after the reduction of mass of nephron. This adaptive hyperfiltration in a single kidney involves a change in renal hemodynamics with an increase in renal blood flow occurring within the days after nephrectomy (22, 23). Glomerular hyperfiltration in the remaining functional nephrons is attributed to injury to the remaining kidney resulting in an increased BP, albuminuria, and ultimately end-stage kidney disease in some donors (19, 24). Even though the absolute value is not clinically relevant, increased proteinuria after the donation was noted.

Previously, the effect of mild isolated kidney decreases in GFR on the biochemical parameters was mostly studied in patients with advanced CKD or undergoing dialysis. Since the disturbance of mineral bone metabolism starts at CKD stage 2 (25, 26), it might be of great interest to study the change of the biochemical parameters in the LKDs, which induce a decrease in the function of kidney with less confounding factors. Experimental data suggest that FGF-23 increase, which targets the remnant nephrons, is an early event in CKD, thereby enhancing renal phosphate excretion and inhibiting the production of 1,25-dihydroxyvitamin D (27). Therefore, FGF-23 is likely an important mediator of the tubular adaptation of phosphate regulation in CKD. We measured FGF-23 in 34 LKDs and analyzed changing pattern following donation. FGF-23 tended to increase in a semi-logarithmic fashion after donation (85.20, IQR: 70.88–386.27 to 106.71, IQR: 73.91–1072.75 pg/ml) (*p* = 0.092) (Supplementary Figure S1). Our results consistently showed that elevation of the phosphaturic hormones, PTH and FGF-23, and decline of serum phosphorus level occurred after mild functional loss of the nephron in the LKDs (10, 28–30).

Despite several reports of mineral bone metabolism disturbance after donation, the predictors of the development of MBD among kidney donors have not yet been investigated. Furthermore, the effect of a modest decrease in GFR on the development of MBD among kidney donors is not clear (31). As FEPi is suggested to be a surrogate marker of phosphate regulation and an alternative to FGF-23 (7), we considered the greater change in FEPi is associated with the development of MBD. The donors with greater change in FEPi had a higher percent decline of eGFR. Functional gain of the remaining kidney and mGFR of the donated kidney *per se* was not associated with the change in mineral bone parameters. However, it was a significant predictor of MBD after donation, when we considered both together. In other words, when the loss of the nephron is large, but the functional gain is small, which induces a high percent decline of eGFR, the risk of MBD is high. Thus, insufficient compensation to GFR reduction in the early predonation period might yield inadequate mineral bone metabolism adaptation.

In addition, preexisting HTN and predonation FEPi were the significant predictors of MBD after donation. Hypertensive nephrosclerosis might cause subtle scarring of the kidney that may not be detected before donation (32) and subclinical pathology may impair compensation after uni-nephrectomy (33). With respect to another predictor, predonation FEPi, due to a tightly regulated process of phosphate reabsorption to maintain phosphate homeostasis (34), phosphate excretion reflects not only the changes in phosphate balance but also dietary intake. However, this study had a limitation in no data on dietary phosphate intake. Despite the limitation, we confirmed that percent decline in eGFR and preexisting HTN are the predictors of greater change in mineral bone metabolism upon adjusting for the impact of predonation of FEPi through multivariable logistic regression.

Our study had several limitations. Comparison with healthy control groups has not been presented and only short-term follow-up data have been shown. However, donor design would be adequate in comparison to short-term outcome associated with predonation demographic or clinical traits (31). Although a recent study showed that kidney donation is associated with an increased risk of ischemic heart disease in long-term follow-up (35), there is limited evidence of how early elevated renal phosphate excretion is associated with vascular calcification, cardiovascular disease, and long-term prognosis after uni-nephrectomy. Long-term follow-up with a healthy control group may be required to clarify this issue. Nevertheless, it is meaningful that this is the first study to investigate the predonation characteristics and physiologic changes, which result in differential mineral bone metabolism adaptation.

In conclusion, this study demonstrated that the LKDs who undergo a mild decline in kidney function without any other comorbidities experience a significant alteration of mineral bone metabolism. Additionally, the change is more significant in the LKDs with preexisting HTN, lower baseline FEPi, and greater decline in eGFR after donation. Therefore, our results suggest that initial workup of mineral bone metabolism parameters is required and postdonation follow-up is needed for the LKDs who had risk factors such as lower baseline FEPi or preexisting HTN.

## Data Availability Statement

The raw data supporting the conclusions of this article will be made available by the authors, without undue reservation.

## Author Contributions

HL participated in designing the study, analyzing and interpreting the data, and writing the manuscript. SHE, EJK, HJC, and CWY participated in collecting the data. BHC participated in designing the study, analyzing and interpreting the data, and revising the manuscript. All authors contributed to the article and approved the submitted version.

## Funding

This study was supported by a grant from the Basic Science Research Program through the National Research Foundation of Korea (NRF), Republic of Korea (NRF-2020R1C1C1008346).

## Conflict of Interest

The authors declare that the research was conducted in the absence of any commercial or financial relationships that could be construed as a potential conflict of interest.

## Publisher's Note

All claims expressed in this article are solely those of the authors and do not necessarily represent those of their affiliated organizations, or those of the publisher, the editors and the reviewers. Any product that may be evaluated in this article, or claim that may be made by its manufacturer, is not guaranteed or endorsed by the publisher.

## Supplementary Material

Supplementary Figure S1 Change in semi-log FGF-23 concentration after donation. 34 donors were used. Median denoted above each bar.

## References

[1] Kovesdy CP Kalantar-Zadeh K. Bone and mineral disorders in pre-dialysis CKD. Int Urol Nephrol. (2008) 40:427–40. 10.1007/s11255-008-9346-7 18368510

[2] Barnato S Sprague SM. Advances in renal bone disease: osteoporosis and chronic kidney disease. Curr Rheumatol Rep. (2009) 11:185–90. 10.1007/s11926-009-0025-1 19604462

[3] Nickolas TL McMahon DJ Shane E. Relationship between moderate to severe kidney disease and hip fracture in the United States. J Am Soc Nephrol. (2006) 17:3223–32. 10.1681/ASN.2005111194 17005938

[4] Kim Y Yoo KD Kim HJ Koh J Yu Y Kwon YJ. Association of serum mineral parameters with mortality in hemodialysis patients: data from the Korean end-stage renal disease registry. Kidney Res Clin Pract. (2018) 37:266–76. 10.23876/j.krcp.2018.37.3.266 30254851 PMC6147195

[5] Vervloet M. Renal and extrarenal effects of fibroblast growth factor 23. Nat Rev Nephrol. (2019) 15:109–20. 10.1038/s41581-018-0087-2 30514976

[6] Oh KH Kang M Kang E Ryu H Han SH Yoo TH. The KNOW-CKD Study: What we have learned about chronic kidney diseases. Kidney Res Clin Pract. (2020) 39:121–35. 10.23876/j.krcp.20.042 32550711 PMC7321679

[7] Hong YA Lim JH Kim MY Kim Y Yang KS Chung BH. Assessment of tubular reabsorption of phosphate as a surrogate marker for phosphate regulation in chronic kidney disease. Clin Exp Nephrol. (2015) 19:208–15. 10.1007/s10157-014-0962-5 24682550

[8] Wolf M. Forging forward with 10 burning questions on FGF23 in kidney disease. J Am Soc Nephrol. (2010) 21:1427–35. 10.1681/ASN.2009121293 20507943

[9] Evenepoel P Naesens M. Mineral metabolism disturbances in kidney donors: smoke, no fire (yet). Kidney Int. (2016) 90:734–6. 10.1016/j.kint.2016.07.001 27633867

[10] Kasiske BL Kumar R Kimmel PL Pesavento TE Kalil RS Kraus ES. Abnormalities in biomarkers of mineral and bone metabolism in kidney donors. Kidney Int. (2016) 90:861–8. 10.1016/j.kint.2016.05.012 27370408 PMC5026566

[11] Levey AS Stevens LA Schmid CH Zhang YL Castro AF 3rd Feldman HI. A new equation to estimate glomerular filtration rate. Ann Intern Med. (2009) 150:604–12. 10.7326/0003-4819-150-9-200905050-00006 19414839 PMC2763564

[12] Walton RJ Bijvoet OL. Nomogram for derivation of renal threshold phosphate concentration. Lancet. (1975) 2:309–10. 10.1016/S0140-6736(75)92736-1 50513

[13] Russell CD Bischoff PG Rowell KL Kontzen F Lloyd LK Tauxe WN. Quality control of Tc-99m DTPA for measurement of glomerular filtration: concise communication. J Nucl Med. (1983) 24:722–7. 6348219

[14] Gates GF. Split renal function testing using Tc-99m DTPA. A rapid technique for determining differential glomerular filtration. Clin Nucl Med. (1983) 8:400–7. 10.1097/00003072-198309000-00003 6357589

[15] Du Bois D Du Bois EF. A formula to estimate the approximate surface area if height and weight be known. Nutrition. (1989) 5:303–11; discussion 12-3. 2520314

[16] Lentine KL Kasiske BL Levey AS Adams PL Alberu J Bakr MA. KDIGO clinical practice guideline on the evaluation and care of living kidney donors. Transplantation. (2017) 101(8S Suppl 1):S1–S109. 10.1097/TP.0000000000001769 28742762 PMC5540357

[17] Young A Storsley L Garg AX Treleaven D Nguan CY Cuerden MS. Health outcomes for living kidney donors with isolated medical abnormalities: a systematic review. Am J Transplant. (2008) 8:1878–90. 10.1111/j.1600-6143.2008.02339.x 18671676

[18] Ratner LE Kavoussi LR Chavin KD Montgomery R. Laparoscopic live donor nephrectomy: technical considerations and allograft vascular length. Transplantation. (1998) 65:1657–8. 10.1097/00007890-199806270-00021 9665087

[19] Garg AX Muirhead N Knoll G Yang RC Prasad GV Thiessen-Philbrook H. Proteinuria and reduced kidney function in living kidney donors: a systematic review, meta-analysis, and meta-regression. Kidney Int. (2006) 70:1801–10. 10.1038/sj.ki.5001819 17003822

[20] Bieniasz M Domagala P Kwiatkowski A Gozdowska J Krzysztof O Kieszek RA. The assessment of residual kidney function after living donor nephrectomy. Transplant Proc. (2009) 41:91–2. 10.1016/j.transproceed.2008.08.160 19249485

[21] Choi KH Yang SC Joo DJ Kim MS Kim YS Kim SI. Clinical assessment of renal function stabilization after living donor nephrectomy. Transplant Proc. (2012) 44:2906–9. 10.1016/j.transproceed.2012.05.086 23194994

[22] Lenihan CR Busque S Derby G Blouch K Myers BD Tan JC. Longitudinal study of living kidney donor glomerular dynamics after nephrectomy. J Clin Invest. (2015) 125:1311–8. 10.1172/JCI78885 25689253 PMC4362245

[23] Srivastava T Hariharan S Alon US McCarthy ET Sharma R El-Meanawy A. Hyperfiltration-mediated injury in the remaining kidney of a transplant donor. Transplantation. (2018) 102:1624–35. 10.1097/TP.0000000000002304 29847501 PMC6153061

[24] Kasiske BL Anderson-Haag TL Duprez DA Kalil RS Kimmel PL Pesavento TE. A prospective controlled study of metabolic and physiologic effects of kidney donation suggests that donors retain stable kidney function over the first nine years. Kidney Int. (2020) 98:168–75. 10.1016/j.kint.2020.01.017 32331703

[25] Levin A Bakris GL Molitch M Smulders M Tian J Williams LA. Prevalence of abnormal serum vitamin D, PTH, calcium, and phosphorus in patients with chronic kidney disease: results of the study to evaluate early kidney disease. Kidney Int. (2007) 71:31–8. 10.1038/sj.ki.5002009 17091124

[26] Ix JH Shlipak MG Wassel CL Whooley MA. Fibroblast growth factor-23 and early decrements in kidney function: the Heart and Soul Study. Nephrol Dial Transplant. (2010) 25:993–7. 10.1093/ndt/gfp699 20037168 PMC2902926

[27] Hasegawa H Nagano N Urakawa I Yamazaki Y Iijima K Fujita T. Direct evidence for a causative role of FGF23 in the abnormal renal phosphate handling and vitamin D metabolism in rats with early-stage chronic kidney disease. Kidney Int. (2010) 78:975–80. 10.1038/ki.2010.313 20844473

[28] Young A Hodsman AB Boudville N Geddes C Gill J Goltzman D. Bone and mineral metabolism and fibroblast growth factor 23 levels after kidney donation. Am J Kidney Dis. (2012) 59:761–9. 10.1053/j.ajkd.2011.09.019 22093959

[29] Kasiske BL Anderson-Haag T Ibrahim HN Pesavento TE Weir MR Nogueira JM. A prospective controlled study of kidney donors: baseline and 6-month follow-up. Am J Kidney Dis. (2013) 62:577–86. 10.1053/j.ajkd.2013.01.027 23523239 PMC3724758

[30] Tan SJ Hewitson TD Hughes PD Holt SG Toussaint ND. Changes in Markers of Mineral Metabolism After Living Kidney Donation. Transplant Direct. (2017) 3:e150. 10.1097/TXD.0000000000000660 28405606 PMC5381743

[31] Lentine KL Segev DL. Understanding and Communicating Medical Risks for Living Kidney Donors: A Matter of Perspective. J Am Soc Nephrol. (2017) 28:12–24. 10.1681/ASN.2016050571 27591246 PMC5198293

[32] Poggio ED Braun WE Davis C. The science of Stewardship: due diligence for kidney donors and kidney function in living kidney donation–evaluation, determinants, and implications for outcomes. Clin J Am Soc Nephrol. (2009) 4:1677–84. 10.2215/CJN.02740409 19713294

[33] Meguid El Nahas A Bello AK. Chronic kidney disease: the global challenge. Lancet. (2005) 365:331–40. 10.1016/S0140-6736(05)17789-7 15664230

[34] Blaine J Chonchol M Levi M. Renal control of calcium, phosphate, and magnesium homeostasis. Clin J Am Soc Nephrol. (2015) 10:1257–72. 10.2215/CJN.09750913 25287933 PMC4491294

[35] Haugen AJ Hallan S Langberg NE Dahle DO Pihlstrom H Birkeland KI. Increased risk of ischemic heart disease after kidney donation. Nephrol Dial Transplant. (2021) 2021:gfab054. 10.1093/ndt/gfab054 33624826 PMC9035350

