# Supplementary figures and images for: Alterations in the Mineral Bone Metabolism of Living Kidney Donors After Uni-Nephrectomy: Prospective Observational Study
Source: Front Med (Lausanne). 2021 Oct 15;8:741944. doi: 10.3389/fmed.2021.741944 (PMC8554047; doi:10.3389/fmed.2021.741944)

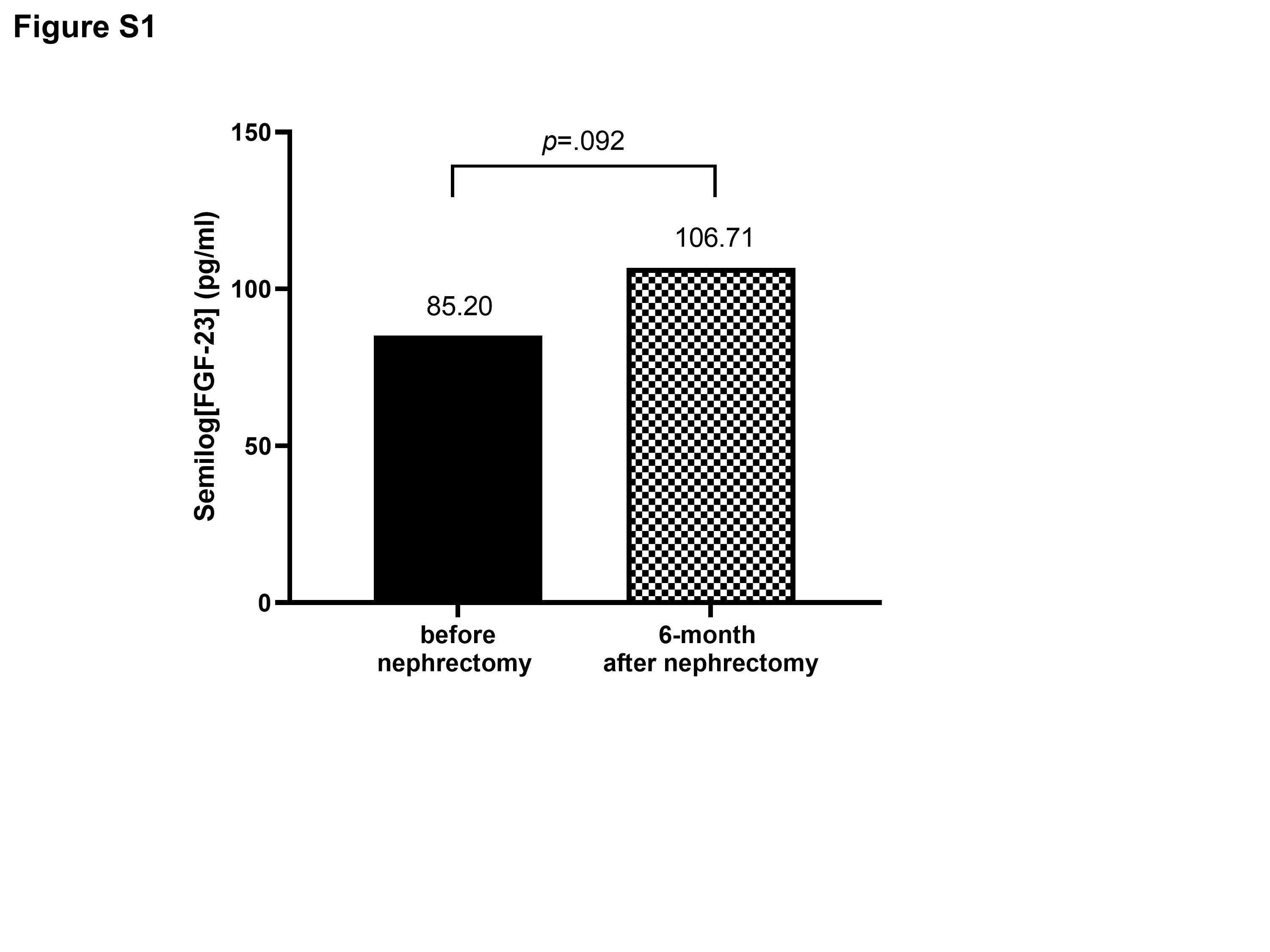

Supplement: Supplementary Figure S1 — Change in semi-log FGF-23 concentration after donation. 34 donors were used. Median denoted above each bar. [file Image_1.JPEG]
